# Probable Vanishing White Matter Disease: A Case Report and Literature Review

**DOI:** 10.4314/ejhs.v31i6.28

**Published:** 2021-11

**Authors:** Endayen Deginet, Robel Tilahun, Solomon Bishaw, Konjit Eshetu, Ayalew Moges

**Affiliations:** 1 Department of pediatrics and child Health, School of Medicine, College of Health Sciences, Dire Dawa University,, Dire Dawa, Ethiopia; 2 Department of pediatrics and child Health, School of Medicine, College of Health Sciences, Haramaya University, Harar, Ethiopia; 3 Department of Radiology, College of Health Sciences, Haramaya University, Harar, Ethiopia; 4 Department of pediatrics and child Health, School of Medicine, College of Health Sciences, Haramaya University, Harar, Ethiopia; 5 Department of Pediatrics and Child Health, School of Medicine, College of Health Sciences, Addis Ababa University, Addis Ababa, Ethiopia

**Keywords:** Vanishing White Matter Disease, Child, Ethiopia

## Abstract

**Background:**

Vanishing white matter disease is one of the most prevalent inherited childhood leukoencephalopathies. The disease is characterized by chronic, progressive and episodic deterioration with ataxia and spasticity.

**Case Presentation:**

Here, we report a 15-month-old female child from Dire-Dawa, eastern part of Ethiopia, who presented with regression of developmental milestones and truncal ataxia since her age of 11 months following a febrile illness that occurred one month earlier. Magnetic resonance imaging of brain is suggestive of vanishing white matter disease.

**Conclusions:**

We believe this case report will increase curiosity, awareness and knowledge of health professionals in Ethiopia and sub-Saharan Africa working with children in early consideration and the diagnosis of the disease.

## Introduction

Vanishing white matter disease (VWMD) is one of the most prevalent inherited childhood leukoencephalopathy. Childhood Leukodystrophies consist of various inherited white matter disorders with an incidence ranging from 1.2 to 3.01 per 100,000 persons per year. The disease is characterized by chronic, progressive and episodic deterioration with ataxia, spasticity and optic atrophy (1, 2).

VWM is caused by mutation in any of the five genes encoding the subunits of eukaryotic translation initiation factor (eIF2B). The disease has an autosomal recessive mode of inheritance (2).

Previously it was known that there is no biochemical marker for this disease, but recently analysis of body fluids has revealed only a few biochemical markers for VWMD. The first marker found was an elevation of cerebrospinal fluid glycine concentrations. A decreased cerebrospinal fluid concentration of asialotransferrin is a recently identified biomarker for VWMD (3, 4). Currently there is no specific treatment for VWMD (2).

Here, we report a 15-month-old female child from Dire-Dawa, eastern part of Ethiopia, who presented with regression of developmental milestones and truncal ataxia since the age of 11 months following a febrile illness that occurred one month earlier. The patient's mother provided informed consent to the publication of this report.

## Case Presentation

We report a 15-month-old female child from Dire-Dawa, eastern part of Ethiopia, first child from non-consanguineous parents who presented with regression of developmental milestones and truncal ataxia since the age of 11 months following a febrile illness that occurred one month earlier. During the initial episode of her febrile illness she was diagnosed to have pneumonia and treated improved. After one month she developed weakness of upper extremity associated with truncal ataxia and difficulty to reach out to objects which was gradually increasing. Up to age of 10 months she had normal growth and development and then she progressively regressed in her developmental milestones. She has no history of seizure, loss of consciousness and prior neurologic illness.

She was born after uncomplicated pregnancy and delivery. She had normal neonatal period and she is immunized according to the EPI schedule.

On physical examination, her vital signs and anthropometric measurements were within normal limit. Head circumference was 48 cm which was within normal limit. She had no Dysmorphic features. On neurologic examination, she was conscious; pupils were midsized and reactive to light bilaterally. Assessment of the optic disc and retina bilaterally had done which was normal. She can move her eyes in all directions. Observed power in all limbs was 3/5. She had bilateral hyperreflexia, truncal ataxia and dysmetria. There is no sensory loss. The laboratory blood work up results was in normal range.

**Figure 1:**
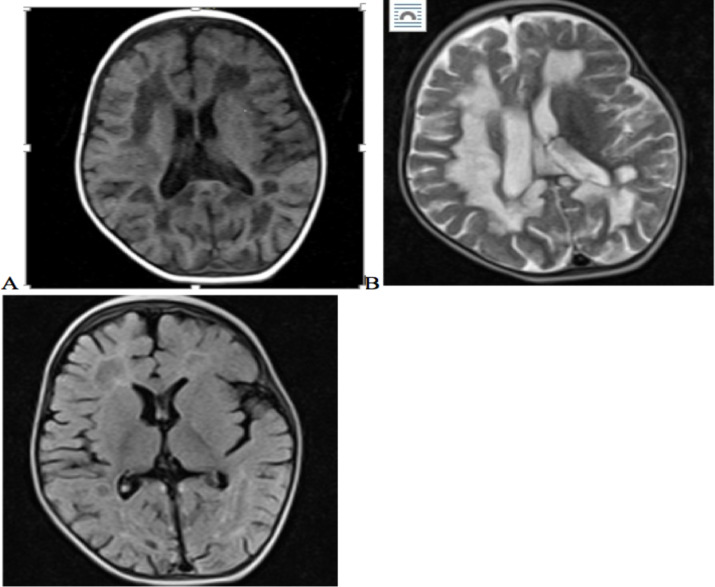
Pre-contrast axial T1-weighted magnetic resonance imaging (**A**) showing diffuse white matter hypo intensity involving the centrum semiovale and corona radiata. Axial T2-weighted magnetic resonance imaging (**B**) showed diffuse hyper intensity. Corpus callosum involvement was seen as hyper intensity; mainly in the splenium. U fibers are spared. Fluid attenuated inversion recovery (FLAIR) images (**C**) showed hypo intense areas, which have signal intensity values close to that of cerebrospinal fluid and were interspersed within the white matter. Basal ganglia, internal capsules, and external capsules were spared.

MRI of her brain revealed diffuse white matter abnormality (Figure 1). Considering history, clinical examinations and investigation reports this case was diagnosed as a case of Vanishing white matter (VWMD) disease.

There is no specific treatment for VWMD; counseling was done with the parents regarding the etiology, progression and outcome of the disease. Advice was given to avoid stressful situations and head trauma and also to take antipyretics and vaccination. She was also advised to have regular follow-up for progression of the condition.

## Discussion

Diagnosis of child hood leukoencephalopathy is based on appropriate clinical history, neuro imaging and molecular genetics. Patients with eIF2B mutations may present with a variety of clinical syndromes. The best-known presentation is the infantile or early childhood presentation with VWM. These children previously healthy present with acute neurologic decompensation after events of physiologic stress, including fever, falls, or fright. The precipitating event is usually followed after several hours by acute motor dysfunction, typically hypotonia or ataxia. The age of the patient and clinical presentation was compatible with our patient. (1)

In our case the patient had chronic progressive neurological abnormal signs such as truncal ataxia, dysmetria and developmental regression after she encountered of first febrile episode which worsened after second episode of febrile illness.

Magnetic resonance imaging has proven to be pivotal in the diagnostic work of patients with leukoencepahlopathies. Special MRI features that are typically seen in a number of specific disorders have a high diagnostic value. White matter rarefaction and cystic degeneration can be visualized best by fluid-attenuated inversion recovery (FLAIR) images. (4).

MRI feature of VWM disease include diffuse signal abnormality of the supratentorial white matter, with less constant signal abnormalities in the cerebellar white matter, brainstem, thalamus, and globus pallidus. In supratentorial white matter, FLAIR images show low signal intensity, isointense with CSF, and suggestive of white matter rarefaction and cystic degeneration. (1, 4, 5). These features are almost consistent with the MRI finding of our case.

Optic atrophy with loss of vision may occur but that was not present in our patient. Genetic testing and biomedical marker test was not available to confirm the diagnosis.

In conclusion, diagnosis of VWMD is made primarily with brain MRI and clinical presentation. There is no cure or specific treatment for VWMD. Avoidance of stressful situations that may trigger deterioration in VWMD is crucial to the management, even though such measures are insufficient to prevent VWMD onset and progression. Since infection and fever can have deleterious effects, antibiotics and antipyretics should be used liberally, and vaccinations, such as the influenza vaccine, should be kept up-to-date.

We believe this case report will increase curiosity, awareness and knowledge of health professionals in Ethiopia and sub-Saharan Africa working with children in early consideration and the diagnosis of VWMD. It will also help as entry point for further research in the above settings since there is no paper published of the subject matter in Ethiopia previously.
